# A Rare Case of Colon Cancer in a Young Patient With HIV Infection: Is it Time for New Colon Cancer Screening Guidelines for HIV Patients?

**DOI:** 10.7759/cureus.43085

**Published:** 2023-08-07

**Authors:** Ahmed Ali Aziz, Muhammad Ali Aziz, Muhammad Humayoun Rashid, Nosheen Omar, Rehan Shah

**Affiliations:** 1 Internal Medicine, Saint Francis Medical Center, Trenton, USA; 2 Internal Medicine, Capital Health Regional Medical Center, Trenton, USA; 3 Internal Medicine, Bronxcare Health System, New York City, USA; 4 Internal Medicine, Capital Health, Trenton, USA; 5 Internal Medicine, Nishtar Medical University, Multan, PAK; 6 Anatomy, University of Health Sciences, Lahore, PAK; 7 Internal Medicine - Rheumatology, Saint Francis Medical Center, Trenton, USA

**Keywords:** gi oncology, cecal mass, screening colonoscopy, colon cancer survillence, hiv and aids

## Abstract

Patients with human immunodeficiency virus (HIV) infection have a higher prevalence of colonic neoplasms than the general population. In these patients, tumors develop at an earlier age, are diagnosed at more advanced stages, and have a dismal prognosis. Current guidelines recommend initiating colon cancer screening in HIV patients at the age of 45 which is consistent with screening age in the general population. We present a rare case of colon cancer diagnosed in an HIV-infected patient at a young age of only 34 years. Therefore, we recommend early screening for colon cancer in HIV patients than the general population.

## Introduction

The advances in the treatment of acquired immune deficiency syndrome (AIDS) / human immunodeficiency virus (HIV) such as the use of highly active antiretroviral therapy (HAART) has significantly increased life expectancy of HIV patients [1]. The HIV patients now carry a lower risk for developing AIDS-defining illnesses and malignancies but are still at risk for developing non-AIDS-defining cancers, such as breast, colon, and prostate neoplasms [1-2]. Colorectal cancer (CRC) is the third most common cancer and the third leading cause of cancer-related deaths in both men and women in the United States [1]. Studies have reported that HIV-infected patients have a higher prevalence of colonic neoplasms than the general population and that in these patients tumors develop at an earlier age, are diagnosed at more advanced stages, and have poor clinical outcomes [1-2] We present a similar case of a young patient with no risk factors for CRC other than history of chronic HIV infection who was diagnosed with colon cancer at a young age of only 34 years.

## Case presentation

**Figure 1:**
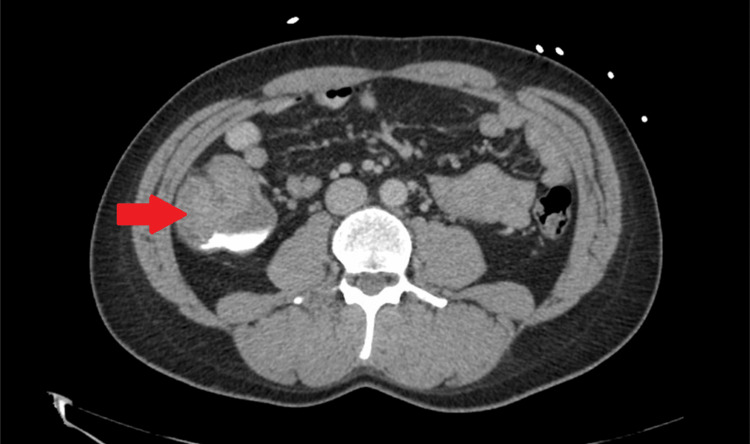
Transverse view of CT scan of the abdomen and pelvis. Red arrow points towards the cecal mass.

**Figure 2:**
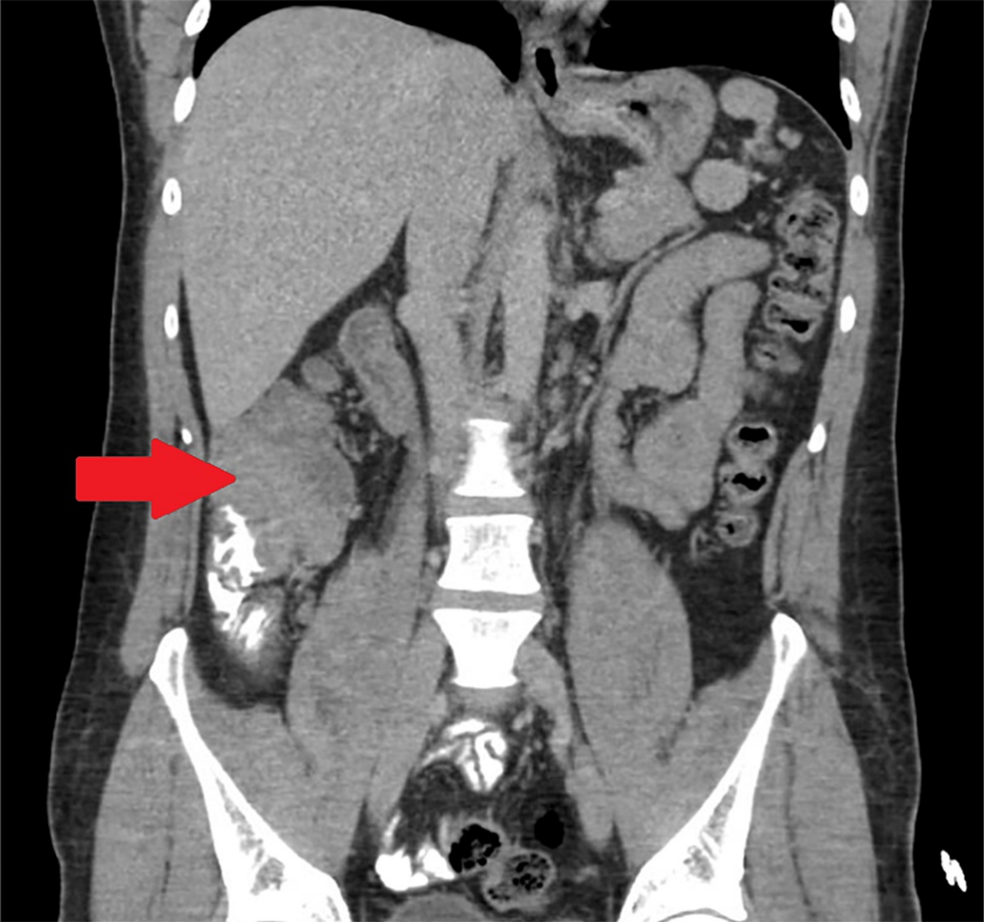
Coronal view of CT scan of the abdomen and pelvis. Red arrow points towards the cecal mass.

A 34-year-old male presented to our emergency department for evaluation of abdominal pain and bloody diarrhea for two days. He also mentioned an unintentional weight loss of 12 pounds over the past month. He had a history of intravenous (IV) drug use and HIV infection diagnosed when he was 25 years old. He was taking bictegravir-emtricitabine-tenofovir alafenamide for the HIV infection and his most recent CD4 count was normal and his HIV viral load was undetectable. He denied any history of malignancy, radiation, or inflammatory bowel disease (IBD) in himself or his family. On presentation his vital signs were stable and within normal limits. On examination, the abdomen was tender to palpation in the right lower quadrant (RLQ). Rectal exam and bowel sounds were normal. Labs were significant for anemia. His hemoglobin was 10.9 g/dL and his hematocrit was 34.3%. White blood cell (WBC) count, platelet count, serum chemistry, coagulation panel, liver function tests (LFTs), and carcinoembryonic antigen (CEA) levels were normal. CT scan of the abdomen and pelvis with IV and oral contrast showed a cecal mass (Figures 1-2).

**Figure 3:**
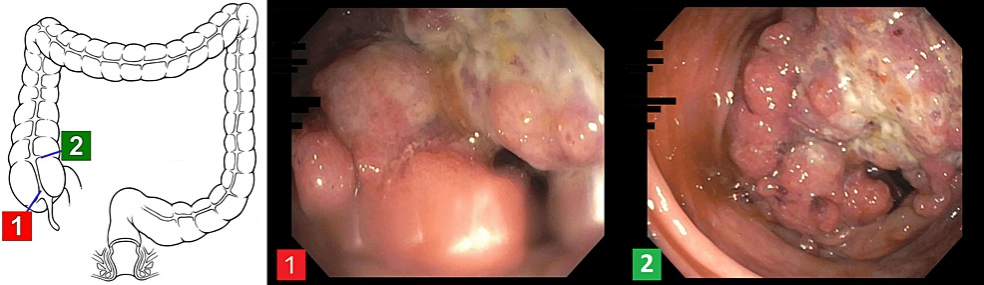
Images of cecal mass as seen during colonoscopy. The numbers "1" and "2" correspond to the location and images of cecal mass in the colon when viewed from the scope camera.

Gastroenterology and General Surgery were consulted for the cecal mass found on imaging. The patient underwent colonoscopy that showed a mass-like lesion in the cecum (Figure 3). Biopsies were positive for adenocarcinoma. The patient underwent right hemicolectomy. Surgical biopsy confirmed Grades 2-3 poorly differentiated adenocarcinoma of the cecum with invasion into the muscularis propria but sparing the pericolonic soft tissue. The tumor stage was T2N0M0. Two days after his surgery the patient was started on clear liquid diet which was later advanced to a regular diet. He was discharged on day 6 of surgery with oncology follow up to initiate genetic counseling and chemotherapy considering his young age.

## Discussion

Our patient presented with symptoms of abdominal pain, bloody diarrhea, and weight loss. The HIV-related colon cancer has been reported to present with weight loss, abdominal pain, change in bowel habits, and bleeding per rectum [3]. Hence, clinicians should consider colon cancer as a differential diagnosis when evaluating HIV patients for these abdominal symptoms, regardless of the patient’s age. 

Our patient had chronic HIV infection and was diagnosed with colon cancer at an age of 34 years. Several studies have reported the increasing incidence of colon cancer being detected in the HIV population at an earlier age with more advanced stage at the time of diagnosis. Bini et al. reported in their study that the prevalence of neoplastic lesions (adenomas or adenocarcinomas) in the colon was significantly higher in HIV-positive patients than in HIV-negative patients (25.5% vs 13.1%) [4]. They also found that HIV-infected subjects were younger and had advanced-stage colon cancers (stage 3 or 4) than control subjects [4]. Iqbal et al. also reported HIV-infected patients to be more likely to have adenomas on screening colonoscopy than non-HIV patients [5]. Wasserberg et al. found that HIV-positive patients were diagnosed with colon cancer at an earlier age (median age 41), had more aggressive presentation, and less favorable outcomes [6]. Since HIV infection predisposes to early development of colon cancer we propose that screening for colon cancer in HIV population should be initiated at an earlier age. Conversely, we also propose that if colon cancer is diagnosed in a young patient; that patient should be screened for HIV as well.

A negative HIV viral load, normal CD4 cell count or being on HAART therapy does not appear to correlate with tumor prevention, tumor grade, tumor stage, or improved outcomes. This is because several factors predispose to the development of CRC in HIV patients such as microsatellite instability [7], HIV oncogenic proteins like the transactivator protein Tat [8], lifestyle factors (including tobacco and alcohol use), immune deficiency, chronic immune activation, and cytokine dysregulation [9]. The HIV oncogenic protein Tat has been reported to be responsible for the younger age of onset and aggressive behavior of colon cancer in HIV patients [8]. Hence, while evaluating a chronic HIV patient with a normal CD4 cell count, negative viral load and good compliance to HAART therapy for abdominal symptoms such as abdominal pain, diarrhea, bleeding per rectum; clinicians should not defer from considering colon cancer as a differential for these symptoms even if HIV infection seems well controlled. 

## Conclusions

Our article raises awareness regarding colon cancer screening in HIV population. We believe that currently colon cancer screening is being underutilized in HIV patient population and may represent a missed opportunity for cancer prevention. Based on our patient's presentation and review of the literature it may be reasonable to begin colon cancer screening earlier in HIV population. It might also be reasonable to correlate colon cancer screening with the chronicity of HIV infection as chronic HIV infection seems to increase the likelihood of colon cancer. The HIV-infected patients presenting with abdominal pain, bloody diarrhea, and weight loss should be evaluated for colon cancer regardless of their age. If colon cancer is detected at a very young age in any patient, we recommend screening for HIV infection in such patients as well. A well-controlled HIV infection should not defer physicians from initiating workup for colon cancer when indicated. 
